# Supplementary material for: A lumenal interrupted helix in human sperm tail microtubules
Source: Sci Rep. 2018 Feb 9;8:2727. doi: 10.1038/s41598-018-21165-8 (PMC5807425; doi:10.1038/s41598-018-21165-8)

A lumenal interrupted helix in human sperm tail microtubules

Davide Zabeo[1], John M. Heumann[2], Cindi L. Schwartz[2], Azusa Suzuki-Shinjo[3], Garry Morgan[2],
Per O. Widlund[4], Johanna L. Höög[1*]

[1] Department of Chemistry and Molecular Biology, University of Gothenburg, Gothenburg 41390, Sweden

[2] Department of Molecular, Cellular and Developmental Biology, University of Colorado, Boulder, CO 80309, USA

[3] Krefting Research Centre, University of Gothenburg, Gothenburg 41390, Sweden

[4] Sahlgrenska Academy, University of Gothenburg, Gothenburg 41390, Sweden

[*] Corresponding author: johanna.hoog@gu.se

Supplementary info:

Supplementary Figure 1: Cryo-EM of the end pieces of four intact human spermatozoa derived from three different donors. A) A cryo-electron micrograph of one sperm tail tip showing repetitive decoration of microtubules (zoomed image). B-D) In all sperm tips the repetitive pattern inside the microtubules in the singlet region is clearly visible. The arrows point to some of the more apparent places, but the helical pattern is present through most, if not all, visible areas.

Supplementary Figure 2: A TAILS-like complex is also present in the terminal parts of the central pair microtubules, around 7.5 micrometers from the sperm tip. A) Slice from a cryo-electron tomogram showing the distal ends of the central pair microtubules (black arrows). B-C) Zoomed-in images of the central pair microtubules show the striations resembling those typical of the TAILs complex.

Supplementary Figure 3: The location of the microtubule seam in single microtubules. A) A top-down view of the 3D model of the microtubule sub-tomogram average, including the TAILS helix. Protofilament numbers have been assigned based on the location of the seam in the dMT A-tubule as in Figure 4F and the numbers correlate to the images in B and C. B) Slices of the sub-tomogram average oriented such that two neighboring protofilaments are visualized. The red cross is the location of the red ball in C). The purple line shows the slope between neighboring tubulin subunits and fits all protofilament pairs except for pair 7-8 where no tubulin subunits could be identified, and pair 8-9 where the slope between protofilaments are different (green

line) showing that the seam is here. C) A longitudinal view of the microtubule 3D model and the protofilaments.

Supplementary movies:

Movie 1: The microtubule singlet region of the intact human spermatozoon. All microtubules have a complete decoration of the TAILS complex. Each frame is a 0.7 nm thick slice from a cryo-electron tomogram. 7 frames/s. Scale bar 100 nm.

Movie 2: Cross-sectional view of the microtubule sub-tomogram average and the electron densities that is the TAILS complex that rotates around the lumen. 6 frames/s. Scale bar 10 nm.

Movie 3: Longitudinal view of the microtubule sub-tomogram average containing the TAILS complex. 6 frames/s. Scale bar 10 nm. 3D model of the sub-tomogram average showing the microtubule lattice in turquoise and the TAILS complex in green. 6 frames/s.

Movie 4: The TAILS complex inside the doublet microtubule. Scale bar 10 nm.

Supplementary Figure 1

Cryo-EM of the end pieces of four intact human spermatozoa derived from three different donors.

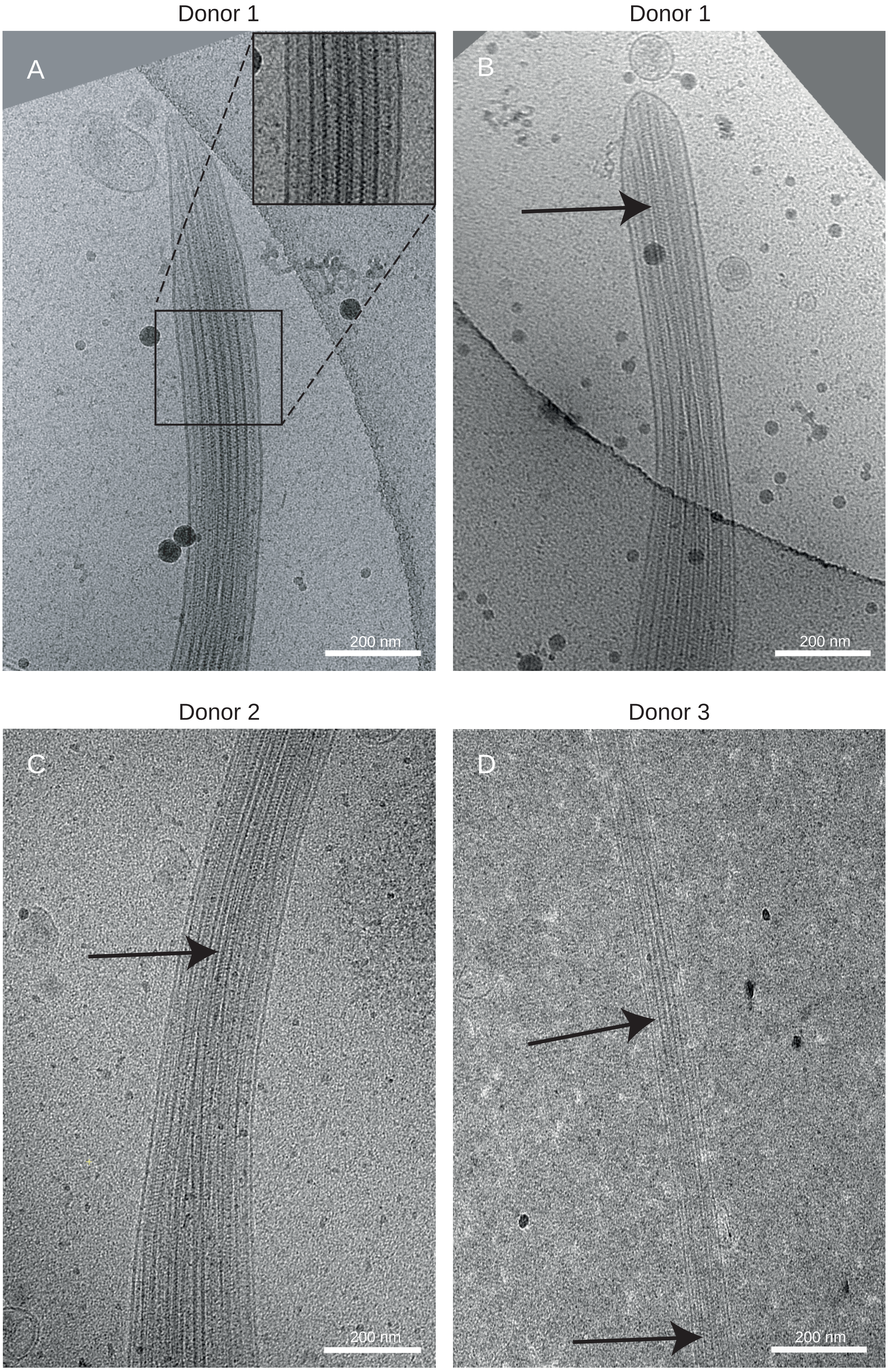

Supplementary Figure 2

A TAILS-like complex is also present in the terminal parts of the central pair microtubules, around 7.5 micrometers from the sperm tip.

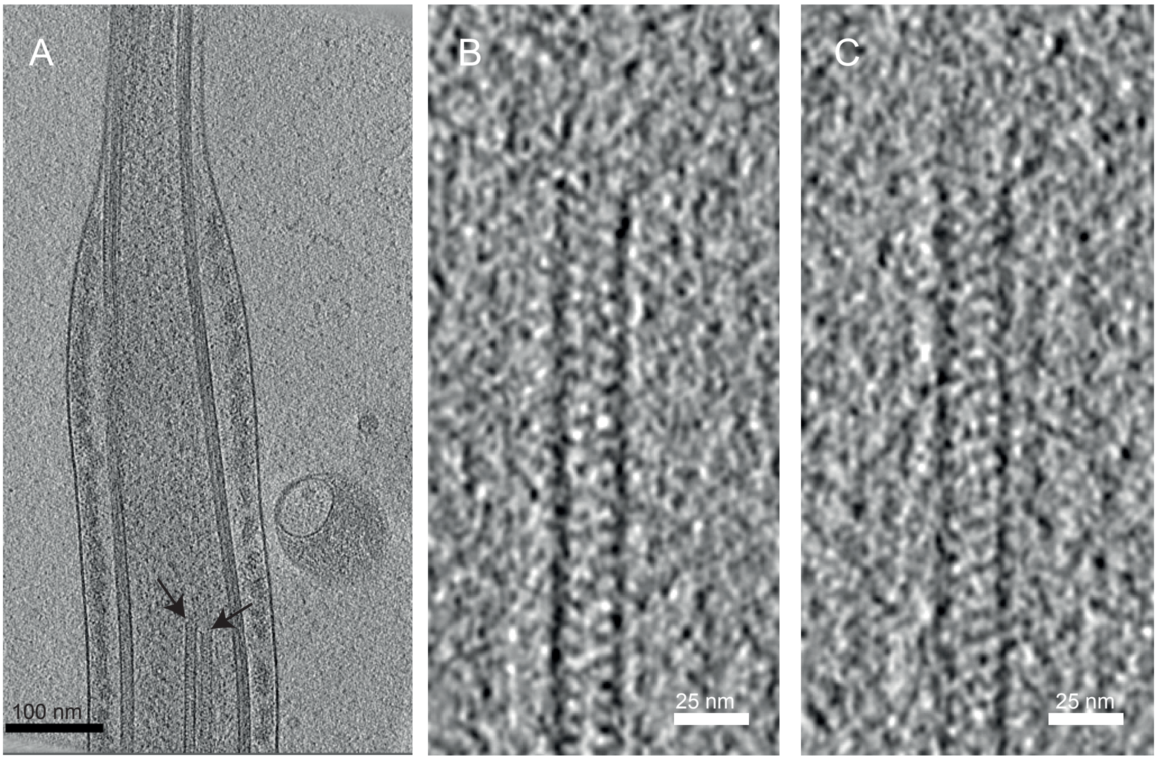

Supplementary Figure 3

The location of the microtubule seam in single microtubules.

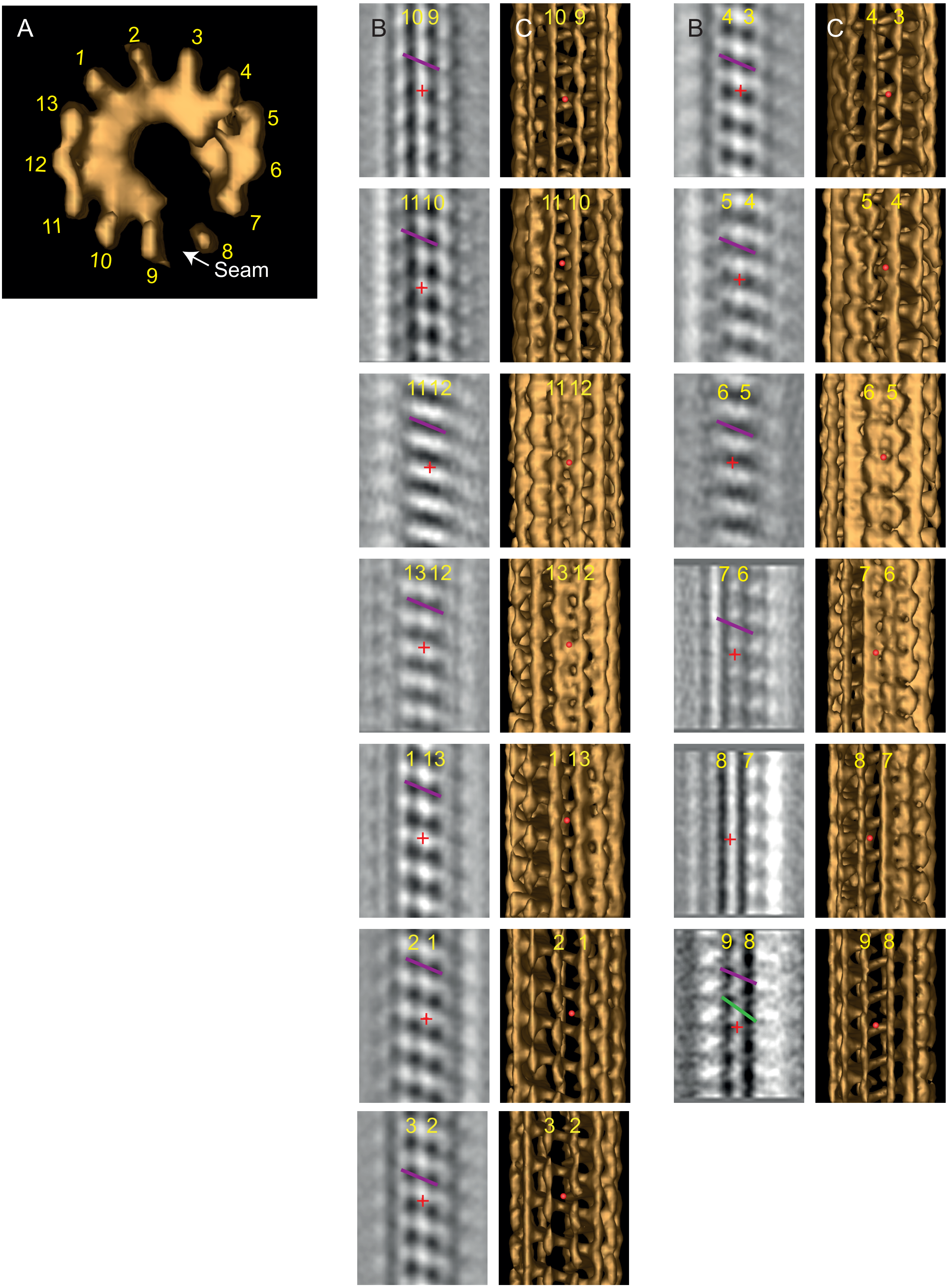

Supplement: Supplementary file 1 — Supplementary Information [file 41598_2018_21165_MOESM1_ESM.pdf]
